# Melanoma Brain Metastases in the Era of Target Therapies: An Overview

**DOI:** 10.3390/cancers12061640

**Published:** 2020-06-21

**Authors:** Paolo Becco, Susanna Gallo, Stefano Poletto, Mirko Pio Manlio Frascione, Luca Crotto, Alessandro Zaccagna, Luca Paruzzo, Daniela Caravelli, Fabrizio Carnevale-Schianca, Massimo Aglietta

**Affiliations:** 1 Istituto di Candiolo, FPO - IRCCS - Str. Prov.le 142, km 3,95, 10060 Candiolo, Italy; paolo.becco@ircc.it (P.B.); stefano.poletto@unito.it (S.P.); Pio.frascione@edu.unito.it (M.P.M.F.); luca.crotto@ircc.it (L.C.); alessandro.zaccagna@ircc.it (A.Z.); luca.paruzzo@ircc.it (L.P.); daniela.caravelli@ircc.it (D.C.); fabrizio.carnevale@ircc.it (F.C.-S.); massimo.aglietta@unito.it (M.A.); 2 Ospedale Mauriziano Umberto I-Largo Turati 62, 10128 Torino, Italy; 3 Department of Oncology, University of Turin, 10124 Torino, Italy

**Keywords:** metastatic melanoma, brain metastases, target therapy

## Abstract

Malignant melanoma is the third most common type of tumor that causes brain metastases. Patients with cerebral involvement have a dismal prognosis and their treatment is an unmet medical need. Brain involvement is a multistep process involving several signaling pathways such as Janus kinase/signal Transducer and Activator of Transcription (JAK/STAT), Phosphoinositide 3-kinase/Protein Kinase B (PI3K/AKT), Vascular Endothelial Growth Factor and Phosphatase and Tensin Homolog (PTEN). Recently therapy that targets the MAPK signaling (BRAF/MEK inhibitors) and immunotherapy (anti-CTLA4 and anti-PD1 agents) have changed the therapeutic approaches to stage IV melanoma. In contrast, there are no solid data about patients with brain metastases, who are usually excluded from clinical trials. Retrospective data showed that BRAF-inhibitors, alone or in combination with MEK-inhibitors have interesting clinical activity in this setting. Prospective data about the combinations of BRAF/MEK inhibitors have been recently published, showing an improved overall response rate. Short intracranial disease control is still a challenge. Several attempts have been made in order to improve it with combinations between local and systemic therapies. Immunotherapy approaches seem to retain promising activity in the treatment of melanoma brain metastasis as showed by the results of clinical trials investigating the combination of anti-CTL4 (Ipilimumab) and anti-PD1(Nivolumab). Studies about the combination or the sequential approach of target therapy and immunotherapy are ongoing, with immature results. Several clinical trials are ongoing trying to explore new approaches in order to overcome tumor resistance. At this moment the correct therapeutic choices for melanoma with intracranial involvement is still a challenge and new strategies are needed.

## 1. Introduction

Melanoma, following breast and lung cancer, is the third most common type of cancer that metastasizes to the central nervous system (CNS). It is estimated that 40–50% of patients with stage IV melanoma eventually develop clinically detectable brain metastases. In autopsy series, over 70% of patients have brain metastasis and a high incidence of subclinical metastasis is noted [1,2,3].

Stage M1d melanoma (CNS involvement) has been associated with a dismal prognosis, with a median overall survival (OS) of 4 months. Many are the predictive factors that negatively impact survival: leptomeningeal involvement, number and size of brain lesions, presence or absence of symptoms and complications and finally, mutational status [4].

For many years chemotherapy and radiotherapy constituted the back-bone of the treatment. Chemotherapy (when used alone) achieved in a small percentage of patients (10%), only a transient control of disease [5,6]. Radiation therapy in the forms of whole-brain radiation (WBRT) was mostly used in symptomatic patients for palliation; more recently more sophisticated forms (stereotactic radiosurgery-SRS or stereotactic radiotherapy-SRT) have been used earlier during the course of the disease, but still they have some limitations (number, size, location of the lesions, burden of disease) that limit their applicability only to a part of patients suffering from brain metastasis.

Since 2011 the prognosis of systemic melanoma has profoundly changed with the introduction of new targeted therapies, as BRAF inhibitors and MEK inhibitors and immunotherapy (anti-CTLA-4 and anti-PD-1). Overall survival (OS) of patients with stage IV melanoma has been significantly improved and now the median OS can reach up to 23 months [7]. Both forms of therapy have a potential impact on the disease also when it has spread to the brain [8].

In this review, firstly we briefly describe the biology, the features of neurotropism and the importance of mutational status. Secondly, we review data about the impact of target therapies, also in combination with radiotherapy, chemotherapy, and immunotherapy, in the populations of patients with stage IV melanoma with brain and leptomeningeal disease, and, last we summarize some new approaches which are still under investigation.

## 2. Biology and Molecular Alterations in Melanoma Brain Metastases

### 2.1. Biology of Cerebral Metastatization

Melanoma brain metastases are frequently the first site of disease-progression [9].

Melanoma CNS invasion is a multistep process [10,11]. Primary tumor cells initially enter the circulation and then undergo hematogenous spread until they arrest within capillary beds of organs, where they proliferate and form the metastasis.

The first step of colonization might be represented by the simple roll of cells on the microvasculature that leads to adhesion to cerebral endothelium [12,13].

Chemokine receptor 4 (CCR4) is one of the most studied molecules implicated in this process and has been proved, from preclinical studies in mouse models, to be over-expressed in melanoma cells colonizing the brain tissues. CCR4 activation leads to increased activity of Phosphoinositide 3-kinase/protein kinase B (PI3K/AKT) pathway [14].

After the adhesion, melanoma cells both secrete serine-proteases which break the endothelial junctions of the blood–brain barrier (BBB) and disrupt its structure and release some other substance as the matrix metalloproteinase-2 (MMP-2) and heparinase [15,16].

These substances allow melanoma cells to infiltrate the BBB, migrate through the endothelium through trans-cellular and para-cellular pathways [17]. After this step, neoplastic cells start feeding with the nutrients from the healthy tissue and proliferate on the surface of the vessels [12]. This process may be facilitated, firstly by sharing the transcriptomic lineage between melanocytes and brain cells and, secondly, by the affinity of melanoma cells for soluble growth factors and cytokines produced within the brain [18]. The microenvironment of the brain, indeed, seems to play an important role in the establishment and maintenance of melanoma metastasis. Astrocyte-secreted factors such as neurotrophins and interleukine (IL-6 and IL-8) have been found to be responsible for influencing the hyperactivation of PI3K/AKT signaling in brain tumor cells [19].

### 2.2. Molecular Alteration in Brain Metastasis

#### 2.2.1. BRAF Mutations

BRAF V600E mutation is observed in 40–50% of metastatic melanomas and drives constitutive activation of MAPK and ERK pathways [20,21]. Paired tissue samples from primary tumors and brain metastases showed an 80% genetic concordance [22]. In 2009, in a murine model, Dankort and colleagues proved how the expression of a constitutively-active BRAFV600E led to a melanoma growth, especially while associated with other altered pathways, such as the inhibition of Phosphatase and Tensin Homolog (PTEN) and the consequent hyper-activation of the PI3K/AKT pathway [23].

#### 2.2.2. PI3K-AKT Pathway

Melanoma brain metastases showed increased expression of the PI3K/AKT pathway compared with the extra-cranial metastases [24]. Activating mutations of AKT or loss of PTEN have an essential role in invasion, cellular migration and adhesion cellular turnover. In melanoma brain metastasis, AKT1 and AKT3 are sites of mutation and the expression of AKTe17k seems to be connected with the higher probability of melanoma brain metastases (MBMs) and shorter survival in the murine model [25].

The cerebral micro-environment is responsible for the activation of AKT by PTEN suppression regardless of BRAF, NRAS or C-kit mutations [19]. Finally, the hyper-activation of the PI3K/AKT pathway could also be implicated in the cell extravasation step [17,26,27,28].

It is possible that interactions between pathways such as MAPK and PI3K/AKT may explain the earlier development of resistance mechanisms in CNS metastases [29,30]. Blocking MAPK may lead to a hyper-activation of the PI3K/AKT stream, which seems to be connected to intracranial metastases growth [31,32,33].

#### 2.2.3. Neoangiogenesis

Melanoma cells obtain nutrients from healthy tissues with the process of neoangiogenesis. Some studies showed that the Janus kinase/signal transducer and activator of transcription (JAK/STAT) pathway is implicated in this process and that STAT3 presents a sustained activation in brain metastasis compared with primary tissues.

Huang and colleagues published in 2006 some evidence that STAT3-dependent signaling played a role in the invasion and new vessel genesis, enhancing the expression of vascular endothelial growth factor (VEGF), Matrix Metallopeptidase2 (MMP2), and fibroblast growth factor 2 (FGF2) [17].

## 3. Target Therapy Approaches

### 3.1. BRAF and MEK Inhibitors for Intracranial Stage IV Melanoma

The introduction of targeted therapies whit BRAF and MEK inhibitors have changed the treatment of stage IV metastatic melanoma. However, patients with active CNS metastases were excluded from most of the clinical trials that led to regulatory approval.

The safety and activity of target therapy in this setting of patients were demonstrated, in the beginning, by retrospective data.

In these studies, the use of a single-agent treatment with BRAF inhibitors, such as Dabrafenib and Vemurafenib, shows an intracranial overall response rate (iORR) ranging between 42% and 50%, an intracranial disease control rate (iDCR) between 66% and 83%, a median progression-free survival (mPFS) ranging between 3.6 and 5.5 months and median overall survival (mOS) ranging between 5.7 and 9.5 months [31,34,35,36,37,38].

The use of combinations of BRAF and MEK inhibitors, as Dabrafenib plus Trametinib, Vemurafenib plus Cobimetinib or Encorafenib plus Binimetinib, allows reaching iORR ranging between 33% and 43%, iDCR between 60% and 79%, a mPFS between 5.3 and 5.8 months and a mOS between 9.5 and 11.2 months [38,39,40].

Recent retrospective data are also available for a new combination of Encorafenib and Binimetinib, published by Holbrook and colleagues in 2019 [40]. In total, 24 patients were enrolled, all of them previously treated. The peculiarity of this study is that 21 of the 24 subjects (88%) treated with the new combination have already received a line with BRAF/MEK inhibitors. In 48% of patients, progression of disease was the cause of treatment interruption. In this specific population retreated with target therapy, a new response of disease was observed in 33% of them with a median duration of response of 22 weeks. These data underlined the possibility of BRAF/MEK inhibitors rechallenge [41,42,43].

Recently, prospective data about the efficacy of BRAF/MEK inhibitors’ associations, specifically in patients with stage IV melanoma and brain involvement, have been published.

The BREAK-MB study enrolled 172 patients with BRAF V600 mutated metastatic melanoma. They were split into 2 cohorts: 89 in cohort A had not received previous local treatment for brain metastases and 83 in cohort B had progressive brain metastases after previous local treatments. The iORR with the use of Dabrafenib single-agent was 39.2% and 30.8%, in cohort A and B, respectively. In patients with a BRAF V600K mutation, the iORR was 6.7% (1 of 15 patients) in cohort A and 22.2% (4 of 18 patients) in cohort B. Among patients with BRAF V600E-mutated melanoma, the mPFS was about 16 weeks in both groups. mOS was 33.1 and 31.4 months, in cohort A and B, respectively. For patients with BRAF V600K-mutated melanoma, the mPFS were 8.1 and 15.9 weeks for cohort A and B, respectively [44].

The activity of Vemurafenib, was evaluated in 146 patients with BRAF V600 mutated melanoma with brain metastases. Similar to the previous study, there was a group of people previously untreated (cohort 1 including 90 patients) and a second group, cohort 2, including 56 patients who were previously treated. The primary endpoint was iORR, showing 18% of responses in both groups. iDCR was again similar, with 61% in cohort 1 and 59% in cohort 2. Median intracranial progression free survival (PFS) was comparable in the two groups: 3.7 months and 4.0 months, respectively. The mOS was 8.9 months in cohort 1 and 9.6 months in cohort 2 [45].

In 2017 the results of the COMBI-MB, a phase 2 clinical trial designed to assess the safety and efficacy of Dabrafenib combined with Trametinib in patients with BRAF V600 mutated melanoma with brain metastases, were published.

Davies et al. treated 125 patients that were divided into four groups. The largest was cohort A, including 76 patients with BRAF V600E mutated melanoma, asymptomatic brain metastasis, ECOG performance status (PS) 0-1 with no previous local brain treatments. In cohort B, 16 patients were included, having the same features as cohort A subjects, but with previous local brain therapies. Asymptomatic subjects (16 patients) with BRAF V600 D, K or R mutated, ECOG-PS 0-1, with or without previous local treatments were regrouped in cohort C. The last 17 patients in cohort D were the symptomatic ones, with an ECOG-PS from 0 to 2.

Leptomeningeal involvement or parenchymal brain metastases greater than 4 cm in diameter were excluded from this study.

Patients in cohort A achieved iORR of 58% (3 Complete response and 41 Partial response), while the iDCR (that included complete and partial responders plus stabilization of intracranial disease) was 78%. Similar results were observed for the extracranial metastases (eORR 55%, eDCR 79%).

In this trial the iORR was 56% in cohort B (9/16 patients), 44% in cohort C (4/16 patients) and 59% in cohort D (10/17 patients), respectively.

Despite these promising intracranial response rates, the median duration of the control of brain disease was only 6.5 months, which was shorter compared with the length of the extracranial response reported of a median of 10.2 months. Among the patients that showed progression of melanoma, in 47% of subjects in cohort A we observed brain disease progression, while in 25% of subjects the disease spread both in intracranial and extracranial sites. Only 9% of patients had isolated extracranial progression.

In cohort A mPFS was 5.6 months and the mOS was 10.8 months. Similar results were also seen in the other cohorts.

The results of this study suggest that intracranial responses in patients with BRAFV600 mutated melanoma were improved by the association of Dabrafenib and Trametinib, compared with the single-agent treatment. However, although the increased response rate observed in this population, the duration was shorter if compared with the results of the previous randomized trial. While subjects with brain metastasis were excluded upfront in phase III trials, the overall response duration, and the mPFS exceeded 12–14 months [29,46,47,48].

No prospective results have been reported to date in patients with stage IV melanoma with brain metastasis treated with other targeted therapies combinations.

Some of the results of the retrospective and prospective trials stated in this chapter have been summarized in Table 1.

At this moment, several studies are ongoing with the aim of improving intracranial response and disease stabilization. For example, the use of higher doses of BRAF/MEK inhibitors is an interesting field inspired by studies of a pulsatile schedule of erlotinib evaluated in non-small cell lung cancer patients with brain metastasis and epidermal growth factor receptor (EGFR) mutation [52].

A new phase 2 trial which randomizes between two-dose regimens of Encorafenib plus Binimetinib in patients with BRAF V600–mutated with brain metastases (NCT03911869) is ongoing. This trial is investigating the impact of an increased dose of Encorafenib (from 450 mg daily to 300 mg twice a day) associated at the standard dose of Binimetinib (45 mg twice a day) and we are waiting for the results.

The MEK-inhibitor Binimetinib also shows modest activity in heavily pretreated patients with NRAS-mutated melanoma with brain metastases in a pilot single-arm study. Between the 11 patients treated in this trial (81% of which were enrolled after failing immunotherapy lines), two had stable disease (SD) and two had partial response (PR) (ORR 18%, DCR 36%) with a median PFS of 2.8 months and a median OS of 6 months [53].

The results reported above show the need for new agents that effectively exert a better intracranial disease control.

An important issue is related to the discontinuation of targeted therapy in those patients who progress extracranially while on BRAF/MEK inhibition. Cagney et al. [54] reported a case series with a dramatically rapid progression of intracranial disease following discontinuation of Dabrafenib and Trametinib because of both extracranial disease progression or toxicity. The authors suggest to closely follow the patients, clinically and radiographically, in the weeks immediately following cessation of BRAFi/MEKi to intercept brain disease progression.

Treatment beyond progression and the association of target therapy and radiation therapy remain possible approaches trying to obtain an intracranial response or, when not possible, a stabilization of the disease.

### 3.2. Target Therapies Combinations with Radiotherapy

Historically, treatment options for melanoma brain metastases were surgery and radiotherapy. Nevertheless, in recent years, target therapy and immunotherapy have radically changed systemic melanoma treatment.

Preclinical findings suggested a therapeutic synergy between Vemurafenib and radiotherapy [55] in particular through an increase in G1 cell cycle arrest in the melanoma cells. Furthermore, radiation therapy could cause a transient disruption in the blood–brain barrier resulting in the uptake of systemic drugs.

Many clinical reports evaluated the efficacy and safety of radiotherapy and BRAF inhibitors in melanoma BRAF V600 mutated patients.

In a retrospective analysis, 12 patients with BRAF V600E mutated melanoma brain metastases were treated with Vemurafenib and radiotherapy (six patients received SRS, six patients received WBRT). Vemurafenib was administered before or during radiation therapy. Symptoms improved in 64% of patients. Of the 48 brain metastases that were treated, 23 (48%) had a complete response and 13 (27%) had a partial response. The median overall survival was 13.7 months. These data suggest a potential synergistic effect in a clinical setting. Two patients died because of cerebral edema. No one experienced neither intracranial bleeding nor G3/4 cutaneous toxicity after therapy [56].

Ahmed et al. treated 24 patients with Vemurafenib and concurrent SRS. The BRAF inhibitor was held 2–3 days before and after SRS treatment. In this study, 12 months-local brain control was 75% and the median overall survival was 7.2 months [57].

Another retrospective study reported the results of 30 patients treated with gamma knife radiosurgery (GNRS) and BRAF inhibitor (Dabrafenib or Vemurafenib). BRAFi was administered before or during GNRS. Median overall survival was 48.8 weeks from the first dose of BRAFi and 24.8 months from the first GNRS. Overall, 78% of patients were alive after 6 months from the beginning BRAFi [58].

Xu et al. have reviewed data from a cohort of 65 patients treated with SRS. Patients were divided into three groups: BRAF-mutated who did not receive target therapy (group A), BRAF-mutated treated with target therapy with Dabrafenib or Vemurafenib (Group B) and BRAF wild type (BRAF-WT) patients (Group C). BRAF-mutated patients treated with SRS and target therapy had a better outcome than other patients. Median OS was 13 months in group B and 1 month in group A and 5 months in group C. Local tumor control rate at 1 year in groups A, B and C was 82.4%, 92%, and 69.2%, respectively [59].

Ly et al. treated 52 patients with melanoma brain metastases with SRS. Seventeen BRAF-mutated patients received BRAFi (concurrent or prior). The authors demonstrated an improved 1-year local control rate for patients treated with SRS and BRAFi compared to SRS alone, without any survival improvement [60].

These studies probably show a benefit by combining radiotherapy and target therapy, but in order to correctly define the therapeutics potential of the combination prospective data are needed.

The toxicity profile of the combination of target therapy and radiotherapy is uncertain. There are some case reports about patients who developed radiation necrosis while Vemurafenib has been started a few days after radiotherapy or during concurrent therapy [61,62].

Patel et al., in a retrospective study, found a statistically significant difference in radiation necrosis between SRS + BRAFi vs. SRS alone [63].

Two studies compared intra-tumor hemorrhage in patients treated with SRT + BRAFi vs. SRT alone with discordant results. The first study [60] found an increased risk of hemorrhage in concurrent treatment versus RT alone, the other study [64] did not find a difference.

These data are inconclusive but recent guidelines recommend holding BRAFi at least 1 day before and after SRS and 3 days before and after radiosurgery. None of these studies report severe skin toxicity.

At this moment, several studies are ongoing, investigating the activity of BRAF/MEK inhibition plus radiotherapy. Between all these trials, the EBRAIN-MEL study (NCT03898908) evaluates the association of Encorafenib plus Binimetinib and local treatments, while RadioCoBRIM trial is investigating the activity of Vemurafenib plus Cobimetinib after radiosurgery (NCT03430947). These trials are active and recruiting at this moment.

Some of the results reported in this chapter have been summarized in Table 2.

### 3.3. Other Approaches to Brain Metastasis

#### 3.3.1. Chemotherapy

Before the targeted therapies development, chemotherapy has been used to exert disease control in the central nervous system. Temozolomide and Fotemustine either as single-agent or in association with radiation therapy were the most common drugs. This choice was related to their ability to cross the brain–blood barrier and reach the brain metastases. Unfortunately, chemotherapy alone has not been able to improve the survival of melanoma patients with CNS involvement, firstly because of its limited systemic activity, and then for the reduced availability in the brain due to the difficult BBB drug penetration [65]. Trials exploring the use of Temozolomide and Lomustine showed an absence of disease control and an mOS of 2 months [66,67,68].

Attempts to improve the activity of chemotherapy have been made by Hwu et al., Hofman et al. and Margolin et al.; these studies investigate the association with radiotherapy (both WBRT and SRT), observing almost the same results with a iORR between 7% and 12% [6,69,70].

One of the few attempts to combine chemotherapy and target therapy has been published by Queirolo and colleagues in 2018. They investigate the possible role of Fotemustine in patients in progression during the BRAF inhibitor Vemurafenib. In the abovementioned study, 31 patients, 16 with brain disease, progressing during Vemurafenib, were treated adding Fotemustine 100 mg/m^2^ infusion every 3 weeks. The overall median PFS was 3.9 months and 61% of patients reached disease control with a median disease control of 6 months. They reported a mOS of 5.8 months. About 16% of patients developed G3/G4 toxicities, and thrombocytopenia was the most commonly reported [71]. At this time chemotherapy remains an option after disease relapse and it could be added to target therapy trying to increase intracranial control of melanoma, but the literature evidence is weak and new trials are needed.

#### 3.3.2. Immunotherapy

Several prospective studies evaluated the use of immunotherapy in patients with intracranial metastatic melanoma. The role of the anti-cytotoxic T-lymphocyte-associated protein 4 (anti-CTLA4) Ipilimumab was evaluated in a phase II study published in 2012 by Margolin and colleagues [72]. They enrolled 51 patients in cohort A (asymptomatic and not receiving corticosteroid) and 21 in cohort B (symptomatic and on a stable dose of corticosteroids), for a total of 72 patients. They found a worse outcome in patients that required steroids; after 12 weeks, 9 patients in cohort A (18%) and only one patient in cohort B (5%) showed disease control. The authors hypothesized that these outcomes could be associated with the different kinds of patients in the cohorts who have a different prognosis but also with the negative effect of steroids on immunotherapy activity.

To improve the efficacy of Ipilimumab, in phase II NIBIT-M1 trial [73] the authors tried to associate Fotemustine, with the hope that chemotherapy-induced release of tumor antigens might amplify Ipilimumab’s antitumor activity. Twenty patients with neurologically asymptomatic brain metastases were treated in this trial, obtaining a disease control in 10 (2 CR, 6 PR, 2 SD). Median PFS was 4.5 months (45% at 1 year) and the median OS was 13.4 months (54.2% at 1 year). An ongoing phase III trial (NIBIT-M2, NCT02460068) is evaluating the efficacy of Fotemustine alone versus the combination of Fotemustine and Ipilimumab or the combination of Ipilimumab and Nivolumab in patients with metastatic melanoma and brain metastasis.

Treatment with anti-PD1 alone was evaluated in a phase II study [74,75]: 23 patients with asymptomatic brain metastases were treated with Pembrolizumab, an anti-PD1 monoclonal antibody. A response in the brain metastasis was achieved in six (26%) patients (2 PR, 4 CR), while the other eight patients were unevaluable for brain metastasis response. There was a median PFS of 2 months and a median OS of 17 months.

To date, the most promising immunotherapy for melanoma patients with brain metastasis is the combination of Nivolumab plus Ipilimumab. Phase II CheckMate 204 trial [8] selected patients with at least one measurable non-irradiated brain metastases and absence of neurologic symptoms to receive the combination Nivolumab plus Ipilimumab. Among the 94 patients evaluable for the primary endpoint, 77% had one or two intracranial target lesions and almost half of the patients had a tumor lesion of 1–2 cm diameter. The rate of intracranial clinical benefit (defined as the percentage of patients who had SD for at least 6 months, CR, or PR) was 57%, with 26% of complete response. The combination prevented intracranial progression for more than 6 months in 64% of patients. Results from a smaller cohort with 18 symptomatic patients, considering whether or not receiving steroids were presented at ASCO 2019, were as follows: the intracranial clinical benefit was 22.2% with an ORR of 16.7% (2 CR, 1 PR, 1 SD), so they confirm the poor prognosis of these kinds of patients [76].

In the Australian ABC trial [77] conducted by Long and colleagues, patients with asymptomatic brain metastases who did not receive previous local brain therapy were randomly assigned to the combination Nivolumab plus Ipilimumab (cohort A, 35 patients) or Nivolumab alone (cohort B, 25 patients), while 16 patients with brain metastases in whom local therapy had failed, or who had neurological symptoms or leptomeningeal disease, received Nivolumab in the non-randomized cohort C. Authors reported intracranial responses in 46% in the combination arm and 20% in the Nivolumab arm. In contrast, only one patient in the unfavorable cohort C had a response. Previous target therapy was permitted, and BRAF and MEK inhibitor treatment-naive patients seemed to have a better response rate.

Some of the results reported in this chapter have been summarized in Table 3.

#### 3.3.3. Combination of Immunotherapy and Target Therapy

In order to understand the possible interactions of BRAF/MEK inhibitor and immunotherapy, several studies have been published. It is well known that oncogenic BRAF can induce immunomodulation leading to an immune-suppressive phenotype that can facilitate the immune escape of melanoma cells. In fact, BRAFV600 mutation increases the production of immunosuppressive factors (IL-10, VEGF, or IL-6), induces the downregulation of MHC class I (MHC-I) molecules, decreases CD8+ T/CD4+ T cell ratio and also the number of NK cells. In contrast, BRAFi treatment can enhance the anti-tumor immunity increasing the expression of immunomodulatory molecules and inducing infiltrating T cells in tumors to express high levels of PD-1 associated with overexpression of PD-L1 in melanoma cells. The addition of MEKi does not seem to impair T-lymphocytes recruitment [78,79].

These findings lead to the design of clinical trials evaluating these combinations, with the aim to improve the outcome of patients with metastatic melanoma, adding the rapid response of the target therapy with the long-lasting response of the immunotherapy. Some preliminary data have been published and confirm a promising activity of these combinations [80,81]

Recently data from the first phase III IMspire150 trial were published [82]. In this trial the combination of Vemurafenib, Cobimetinib and Atezolizumab compared with Vemurafenib, Cobimetinib, and placebo showed a similar overall response rate but significantly prolonged duration in favor of the triplet combination (21.0 vs. 12.6 months, respectively) and this translated to a significant improvement in investigator-assessed progression-free survival (15.1 vs. 10.6 months). Data about the subgroup of patients with intracranial disease are not available at this time. We are also waiting for the results of another ongoing phase II study, evaluating the combination of Vemurafenib and Cobimetinib plus Atezolizumab specifically in patients with brain metastases. (NCT03625141).

## 4. Leptomeningeal Involvement

Leptomeningeal metastases of melanoma have a really unfortunate prognosis with survival of a few weeks [83]. During the last years, even with the notable improvement in the outcomes in melanoma patients, the survival of people with leptomeningeal involvement has been still measured in weeks [84]. In the new era of target therapies, we know that BRAF/MEK inhibitors treatments could pass the BBB, but, what is still not completely sure, is if the drugs reach therapeutic concentrations [85]. The patient-to-patient variability in plasma and cerebrospinal fluid (CSF) level could be due to different BBB integrity, especially after previous local treatments such as surgery or radiotherapy.

While the activity of BRAF inhibitors in brain metastases is well known, few clinical cases are present in the literature about the activity in leptomeningeal disease, describing a modest, but sometimes rapid, clinical symptoms improvement [86,87,88,89]. In the few cases described, some patients could reach a median overall survival of 7.2 months [86]. Scattered data are available about the activity of the associations between anti-BRAF and anti-MEK therapies or combinations of target and immunotherapies, but some pieces of evidence point to the fact that the duration of disease control in the brain and in leptomeningeal metastasis, is shorter compared to the one in extracranial metastasis [29].

The mechanisms of escape from BRAF inhibition have been intensively studied [90]. Some evidence describes how BRAF inhibitors could have less access to the brain and leptomeningeal lesions because of active drug efflux transporters [91]. Additionally, the limited penetration of the blood–brain barrier of BRAF/MEK inhibitors on meninx could be because they have a relatively large molecular weight and poor lipid solubility [92]. Other studies suggest that the inflammatory state of the BBB caused by previous local treatment could be implicated in target therapies penetration and, consequently, lead to reduced activity on the brain and leptomeningeal metastases.

WBRT is used especially when the patient has neurological symptoms, reducing tumor bulk and the obstructive hydrocephalus when it is present. The synergy between radiation and immunotherapy treatments for brain metastasis is also a new field of researches with some studies ongoing. (NCT 03719768) [93]. Almost all the trials with the new checkpoint inhibitors excluded patients with leptomeningeal involvement. There are few anecdotal cases which describe some responses treating patients with anti-CTL4 and anti-PD1 [94,95].

A new exciting approach seems to be the intrathecal administration of drugs known to be effective in metastatic melanoma. At ASCO 2020, the results of a phase I/IB trial with intrathecal administration of Nivolumab will be presented [96].

## 5. Drug-Resistance Mechanisms and the Role of the Micro-Environment

Targeted therapy resistance in melanoma cells has been studied extensively and several mechanisms have been found to be responsible [97].

Johnson et al., in 2015, published a study in which they have analyzed tumor samples of 132 patients progressed under targeted therapies [98]. They detected that NRAS mutations, BRAF splice variants and MEK1/2 mutations tended to occur alone, while others resistance mechanisms, such as BRAFV600 amplifications and other non-MAPK-related alterations commonly arouse concomitantly. In this study they also suggested that, while some of these alterations occurred in a mutually exclusive way, others could operate in complementary ways to drive melanoma drug resistance.

In a review published by Welsh et al. in 2016, the authors extensively studied those different resistance pathways and also resume the steps that research has been made in order to overcome them [99]. Firstly, because of the presence of these acquired and intrinsic mutations, which lead to the reactivation of ERK signaling, ERK has become a target in order to overcome resistance to anti-BRAF targeted therapy. The inhibition of ERK has been evaluated as treatment alone or in combination with other drugs, both in the same MAPK pathway and associated with the suppression of parallel pathways such as PI3K, since ERK inhibition could cause a paradox RAS and PI3K activation which can lead to melanoma resistance [100,101,102].

Secondly alternative survival pathways have been analyzed, especially the PI3K-Akt-mTOR, which lead to drug resistance via mutations in Akt and loss of function PTEN. We saw in previously, how this stream of mutations could be important in melanoma brain diffusion [103,104].

The interconnections between target therapies and the immune system have been largely explored, as previously discussed [105,106].

In the setting of brain metastasis, several pieces of evidence underlined the fact that the targeted therapies have poor permeability across the BBB and this could lead to a sub-therapeutic concentrations which could lead easily to the development of drug resistance mechanism mentioned above [91,107,108].

Recently, the role of the tumor microenvironment (TME), in addition to the previously cited molecular changes in melanoma cells, has been considered part of the mechanisms for drug resistance [109]. Tumor cells use the so-called “aerobic glycolysis” in the proliferation process [110,111]. When oxygen tension reduces, a hypoxic microenvironment develops which assists an upregulation of genes involved in the anaerobic glycolysis pathway. So, lactate and protons are produced in large amounts and transported outside causing acidification of the extracellular pH. This acidity is also sustained by poor blood perfusion and limited lymphatic vessels [112].

Recently a study conducted by Ruzzolini et al., shows, in a preclinical model, that melanoma cells exposed to an acidic medium are resistant to BRAFi and MEKi. It has also been proved that cells acquired traits compatible with an epithelial-mesenchymal transition and showed high resistance to apoptosis. In melanoma culture grown in acidic medium, m-Tor signaling is active and preclinical data suggest that everolimus could be used to overcome target therapy resistance [113]. In addition, the cellular components of TME have a major role in drug resistance. Cancer-associated fibroblasts (CAF) are one of the multiple cells types enriching the tumor microenvironment. CAFs do not only support tumor cells growth and diffusion [114,115,116] but they also have a role in target therapy resistance. Resistance pathways mediated by CAF’s comprehend cellular mediators such as Hepatocyte growth factor (HGF) [117] and Neuregulin1 (NRG1) [118] and they are able to remodel the extracellular matrix (ECM). Under the pharmacological pressure of BRAFi, CAFs create a tumor niche that is rich in ECM proteins, which accelerates the acquisition of drug resistance [119,120,121,122].

## 6. New Strategies to Overcome Melanoma Cells Resistance

New target therapies recently on study are directed against the main mechanism involved in cerebral metastatization and resistance to BRAF and MEK inhibitors. Here we describe some of the most interesting approaches.

### 6.1. PI3K/AKT Pathway

As previously explained, alterations in the PI3K/AKT pathway could be responsible for the resistance to BRAF and MEK inhibitors. Two phase I trials combining BKM120, an orally bioavailable pan-class I PI3K inhibitor, and Vemurafenib in advanced melanoma showed only modest clinical activity with poor tolerability [123,124].

Recently, a new clinical trial evaluating the combination of the AKT inhibitor GSK2141795 with Dabrafenib and Trametinib has completed accrual (NCT01902173) and we are waiting for the results. New studies with isoform-specific PI3K inhibitors are ongoing. Another interesting clinical trial combining PI3K-Beta Inhibitor (GSK2636771) with Pembrolizumab in patients with PTEN loss is now recruiting. Unfortunately, access to these trials for patients with CNS disease is limited to people with stable brain lesions and no leptomeningeal involvement (NCT03131908).

Other pieces of evidence suggest that PI3K pathways could be activated from a different extracellular signal, for example, VEGF [125]. On this basis trials combining Bevacizumab with other agents are recruiting as NCT02681549 (Bevacizumab plus Pembrolizumab in melanoma M1d), or NCT03175432 (Bevacizumab associated with Atezolizumab with or without Cobimetinib in patients with untreated CNS involvement). A clinical trial associating Bevacizumab and Vemurafenib firstly, then emendated with the associations of Cobimetinib, has finished its slow recruitment and we are waiting for its results (NCT01495988).

### 6.2. Cyclin-Dependent Kinase 4 (CDK4) and 6 (CD4-CDK6)

Data regarding the role of Cyclin-dependent serine/threonine kinases on the development of brain metastases are based mainly on studies on breast cancer. Flaherty et al. [126] showed that loss of CDKN2A, that causes p16 loss, correlates with worse outcomes in patients treated with Dabrafenib and Trametinib. The loss of p16 causes the activation of CDK4. Previous in vitro evidence showed that loss of p16 correlated with responsiveness to CDK4 inhibitors [127,128]. CDK inhibitors, such as Ribociclib and Palbociclib, have achieved regulatory approval in other diseases, such as breast carcinoma. At this moment, these molecules are investigated in several clinical trials for patients with metastatic melanoma alone or in combination with BRAF and/or MEK inhibitors [129] (NCT02065063, NCT01781572).

### 6.3. CXCR4 Antagonist

Klein et al. show that CCR4 expression is associated with increased development of brain melanoma metastases [14].

X4P-001(AMD3100) is an orally bioavailable CXCR4 antagonist that has demonstrated activity in various tumor models. CXCR4 is the receptor for CXCL12, which has potent chemotactic activity for lymphocytes and MDSCs (myeloid-derived suppressor cells). The use of CXCR4 antagonist has the role of unmasking the tumor to immune attack by multiple mechanisms, such as decreasing the infiltration of MDSCs, increasing the ratio of CD8+ T cells to Treg cells and eliminating tumor revascularization. Because of this evidence, a clinical trial of the combination of X4P-001 and Pembrolizumab in patients with advanced melanoma has been conducted. The administration of a CXCR4 antagonist (AMD3100) induced rapid T-cell accumulation among the cancer cells and, in combination with anti-PD-L1, synergistically decreased tumor growth. (NCT02823405). The results of this interesting approach are not published yet.

### 6.4. Other Approaches

While mutations of BRAF and NRAS are the most commonly described in melanoma cells, a number of less common aberrations have been identified, which might also provide new therapeutic opportunities and identification of new targets continue to increase therapeutic options.

Data from preclinical models show that MBMs use mitochondrial oxidative phosphorylation (OXPHOS) more than extracranial metastases and primary melanomas. An OXPHOS inhibitor, IACS-010759, prolonged survival in mice with intracranial melanoma xenografts. OXPHOS enrichment in MBMs may have a role in the resistance to MAPK-directed target therapies observed in patients with brain involvement [24]. Although preclinical data show that single-agent IACS-010759 have some beneficial effects, future studies should assess the efficacy and safety of a combination of this new molecule and BRAFi plus/or MEKi targeted therapies. Murine model experiments suggest that the use of IACS-010759 may also reduce the risk of brain metastasis development and prevent resistance to MAPKi therapy [130,131]. The combination of MAPKi therapy and IACS-010759 could effectively change the story of the disease by reducing the incidence of CNS invasion and the incidence of MAPKi resistance.

Leczano et al. identify that Neurotrophic tropomyosin receptor kinase (NTRK) rearrangement in metastatic non-acral cutaneous melanoma is less than 1% [132]. Recent results have been reported about the efficacy and safety in solid tumors with brain metastases of Entrectenib, an inhibitor of kinases which is encoded by the NTRK gene [133], while a new study is enrolling patients also with melanoma in a new clinical trial, the basket study STARTRK-s (NCT02568267).

## 7. Conclusions

Patients with metastatic melanoma with CNS involvement still have a worse prognosis compared with patients with the extracranial disease. Recently several steps forward have been made with the introduction of target therapies, which have improved disease control and overall survival also in patients with brain involvement. Unfortunately, intracranial disease seems to be less sensitive to the available treatments and it can quickly develop mechanisms of resistance that are challenging to overcome. Combinations of systemic treatments with local approaches such as radiotherapy as well as the association of target therapy drugs with the new immunotherapy monoclonal antibody could try to overcome some mechanism of resistance and allow better control of the disease. Leptomeningeal involvement is another big challenge. Since the beginning, patients with this particular site of melanoma invasion had the worst prognosis due to the peculiarity of the meninx tissue and the difficulty in reaching the site by drugs. In order to improve the clinical outcome of patients with CNS involvement, new molecules are under investigation and many are the targets considered (from neoangiogenesis to metabolic pathways); their biological basis is robust and the preliminary results are encouraging but still far from a direct clinical translation into daily practice. The high incidence of CNS involvement in melanoma patients must be considered by the oncological community as an important issue to be addressed. Only the continued cooperation between the different branches of Oncology will allow the improvement of the survival and the quality of life of melanoma patients with brain disease.

## Figures and Tables

**Table 1:** Study of BRAF and MEK inhibitors in melanoma with brain metastases.

Study	Trial Design	Drug(s)	No. Patients	IORR % (CR+PR)	IDCR % (CR+PR+SD)	Median PFS (Months)	Median OS (Months)
Gorka E. (2018) [34]	retrospective	Dabrafenib	30	43	83	5.5	8.8
Dzienis M.R. (2014) [35]	retrospective	Vemurafenib	22	50	NA	NA	NA
Harding J.J. (2015) [31]	retrospective	Vemurafenib	22	50	82	4.1	7.5
Gibney G.T. (2015) [36]	retrospective	Vemurafenib	283	48.1	67.2	NA	59% at 12 months
Martin- Algarra S. (2019) [37]	retrospective	Dabrafenib	132	NA	NA	3.9	9.5
Geukes Foppen M.H. (2018) [38]	retrospective	Dabrafenib	31	NA	NA	5.7	8.8
		Vemurafenib	85	NA	NA	3.6	5.7
		Dabrafenib + Trametinib	30	NA	NA	5.8	11.2
Drago J.Z. (2019) [39]	retrospective	Dabrafenib + Trametinib, Vemurafenib + Cobimetinib, Encorafenib + Binimetinib, Vemurafenib + Trametinib	65	NA	NA	5.3	9.5
Holbrook K. (2020) [40]	retrospective	Encorafenib + Binimetinib	24	33	79	NA	NA
Davies M.A. (2017) COMBI-MB [29]	Phase II	Dabrafenib + Trametinib	125				
		cohort A	76	58	78	5.6	10.8
		cohort B	16	56	88	7.2	24.3
		cohort C	16	44	75	4.2	10.1
		cohort D	17	59	82	5.5	11.5
Dummer R. (2014) [49]	Pilot Study	Vemurafenib	24	16	84	3.9	5.3
Long G.V. (2012) BREAK-MB [44]	Phase II	Dabrafenib	172				
		cohort A	74	39.2	81.1	16.1	33.1
		cohort B	15	6.7	33.3	8.1	16.3
		cohort C	65	30.8	89.2	16.6	31.4
		cohort D	18	22.2	50	15.9	21.9
Falchook G.S. (2012) [50]	Phase II	Dabrafenib	10	NA	90	4.2	NA
McArthur G.A. (2017) [45]	Phase II	Vemurafenib	146				
		cohort A	90	18	61	3.7	8.9
		cohort B	56	18	59	4.0	9.6
Arance A.M. (2016) [51]	Phase III	Vemurafenib	66	18	NA	NA	NA

IORR: intracranial overall response rate; IDCR: intracranial disease control rate; CR: complete response; PR: partial response; SD: stable disease; PFS: progression free survival; OS: overall survival; NA: not available.

**Table 2:** Study of targeted therapy associated with radiation therapy in melanoma with brain metastases.

Study	Trial Design	Drug(s)	RT	No. Patients	Adverse Events	Efficacy Data	Survival Results
Narayana A. (2013) [56]	retrospective	Vemurafenib	SRS- WBRT	12	4 intratumoral bleed prior to therapy. 2 patients developed steroid dependence. 1 radiation necrosis. 2 deaths for cerebral edema.	Symptoms improved in 64%. Radiographic responses in 36/48 (75%) Responses of index lesions (48% CR; 27% PR)	Median OS 13.7 (months)
Ahmed K.A. (2015) [57]	retrospective	Vemurafenib	SRS	24	1 radiation necrosis	75% local control at 12 months	Median OS 7.2 (months)
Gaudy- Marqueste C. (2014) [58]	retrospective	Dabrafenib or Vemurafenib	GNRS	30	3 Radiation necrosis. 3 edemas and 3 hemorrhages within 2 months after GNRS; 4 edemas and 7 hemorrhages later	Median OS from first GNRS under BRAF-I and first dose of BRAF-I: 24.8 and 48.8 weeks, respectively	NA
Xu Z. (2017) [59]	retrospective	Dabrafenib or Vemurafenib	SRS	17	NA	Local tumor control rate at 1 year 92%	Median OS 13 (months)
Ly D. (2015) [60]	retrospective	Dabrafenib or Vemurafenib	SRS	17	39.3% free from intratumoral hemorrhage at 1 year	Local control rate 85% at 1 year	1year-OS 50.2%
Patel K.R. (2016) [63]	retrospective	Dabrafenib or Vemurafenib	SRS	15	Radiation necrosis (radiographic) 22.2% at 1 year; Radiation necrosis (symptomatic) 28.2% at 1 year	1year-local failure 3.3% 1year-distant intracranial failure 63.9%	1year-OS 64.3%

SRS: stereotactic radiosurgery; WBRT: whole brain radiotherapy; GNRS: gamma knife radiosurgery; NA: not available; CR: complete response; PR: partial response; OS: overall survival.

**Table 3:** Prospective study of Immunotherapy in melanoma with brain metastases.

Study	Trial Design	Drug(s)	No. Patients	IORR % (CR+PR)	IDCR (CR+PR+SD)	Median PFS (Months)	Median OS (Months)
Margolin K. (2012) [72]	Phase II	Ipilimumab	72				
		Cohort A	51	16	24	2.7	7.0
		Cohort B	21	5	10	1.3	3.7
Di Giacomo A.M. (2012) NIBIT-M1 [73]	Phase II	Ipilimumab + Fotemustine	20	40	50	4.5	13.4
Goldberg S.B. (2016) [74]	Phase II	Pembrolizumab	23	26	30.4	2	17
Long G.V. (2018) ABC [77]	Phase II	Nivolumab + Ipilimumab					
		Cohort A	35	46	57	NR	NR
		Nivoluamb					
		Cohort B	25	20	20	2.5	18.5
		Cohort C	16	6	18	23	5.1
Tawbi H.A. (2018) Checkmate204 [8]	Phase II	Nivolumab + Ipilimumab	94	55	57	NR (56.6% at 12 months)	NR (81.5% at 12 months)

IORR: intracranial overall response rate; IDCR: intracranial disease control rate; CR: complete response; PR: partial response; SD: stable disease; PFS: progression free survival; OS: overall survival; NR: not reached.
